# Impact of urban density on human well-being and sustainable development in Delhi, India

**DOI:** 10.1038/s41598-025-16033-1

**Published:** 2025-09-30

**Authors:** Ram Avtar, Sohail Ahmad, Md. Mustafizur Rahman, Saleh Alsulamy, Gowhar Meraj, Mahendra Sethi, Chander K. Singh, Ali Kharrazi

**Affiliations:** 1 Faculty of Environmental Earth Science, Hokkaido University, Sapporo, Japan; 2 Department of Civil Engineering, Chennai Institute of Technology, Chennai, Tamil Nadu, 600069 India; 3 School of Computing, Engineering and Physical Sciences, University of the West of Scotland, Paisley, PA1 2BE UK; 4 Capital City Development Authority (RAJUK), Dhaka, 216-2035 Bangladesh; 5 Architecture and Planning Department, College of Engineering, King Khalid University, Abha, 61421 Kingdom of Saudi Arabia; 6 Department of Biology, Chemistry and Environmental Sciences, College of Arts and Sciences, American University of Sharjah, 26666, Sharjah, United Arab Emirates UAE; 7 Indian Society for Applied Research and Development, New Delhi, India; 8 Department of Natural and Applied Sciences, TERI School of Advanced Studies, New Delhi, 110070 India; 9 Advancing Systems Analysis (ASA) program, International Institute for Applied Systems Analysis, Schlossplatz 1, Laxenburg, A-2361 Austria; 10 Network for Education and Research on Peace and Sustainability (NERPS), Hiroshima University, Higashihiroshima, Japan; 11 College of Management and Economics, Tianjin University, Tianjin, 300072 China; 12 Department of Environmental Science, School of Vocational Studies and Applied Sciences,, Gautam Buddha University, Greater Noida, Uttar Pradesh, 201312 India

**Keywords:** Urban density, Neighborhoods, Human well-being, Suburban planning, Delhi, Environmental sciences, Environmental impact

## Abstract

Achieving sustainable urban development amid rapid urbanization requires a deep understanding of how urban density influences human well-being. This study examines the spatial relationship between built-up population density (BUD) and human well-being across Delhi, one of the world’s fastest-growing megacities. Using a combination of high-resolution census data, remote sensing, and spatial analysis, the study identifies markedly uneven urban form characterized by extreme density variation, ranging from 2,884 to 136,385 persons per km² across clusters, and uncoordinated development, particularly in peripheral zones. While BUD significantly affects well-being outcomes, the analysis reveals that beyond an optimal threshold, socio-economic conditions become equally influential. The findings emphasize the urgent need for differential planning strategies: promoting infrastructure and planned densification in low-density peripheries; encouraging mixed-use development in moderate-density zones; and alleviating congestion while enhancing services in high-density, unplanned areas. These insights provide a policy framework aligned with the Sustainable Development Goals, particularly Goal 11, which aims to make cities inclusive, safe, resilient, and sustainable. By emphasizing the spatial heterogeneity of urban density and its implications for well-being, this research provides a valuable lens for urban policy and planning in rapidly growing global cities.

## Introduction

Urbanization has become a defining global trend, driven by population growth, economic development, and rural-to-urban migration^1^. As cities expand, built-up areas grow rapidly, bringing complex implications for environmental sustainability, infrastructure accessibility, and human well-being^2^. Urban densification is often promoted as a pathway to sustainable development; however, its impacts on quality of life remain contested. In alignment with the Sustainable Development Goals (SDGs), particularly SDG 11, policymakers increasingly emphasize the need for cities to be inclusive, safe, resilient, and sustainable^3,4^. The Sustainable Livelihood Security (SLS) approach has been applied to urban contexts in India to assess development priorities and sustainability dimensions^5^.

Within this broader discourse, Built-up Urban Density (BUD) emerges as both a driver and outcome of urban dynamics – shaped by and shaping socio-economic, political and spatial conditions. This dual perspective highlights the intricate linkages between urban form and human well-being, offering a holistic lens through which to assess sustainability in urban environments. Recent studies emphasize the spatial heterogeneity of urban environments and their influence on vulnerability and environmental quality. For example, GIS-based models have been employed to assess urban social vulnerability in Eastern India, revealing multiscale patterns of risks and inequality^6^.

However, a significant research gap remains regarding how BUD varies at intra-city scales and how these variations directly influence human well-being. Much of the existing literature on urban density is focused at the macro levels - evaluating citywide averages or inter-city comparisons, often with limited attention to neighborhood-level patterns. Studies using logistic regression to simulate urban expansion in Delhi have revealed key drivers of spatial growth^7^, while others have used GIS based Multi-Criteria Decision Analysis (MCDA) techniques to assess the environmental quality and waterlogging risk^8^. Exploratory spatial analyses have provided new insights into urban forms in Class-I cities^9^, and research on urban morphology has emphasized the influence of spatial variables such as vegetation, street connectivity, and commercial activity on quality of life^10^. Methods like Geographically Weighted Principal Component Analysis (GWPCA) have further advanced spatial analysis studies of urban vitality^11^. Nevertheless, these efforts often fall short of addressing micro-scale density variations and their implications for sustainable urban planning.

This study aims to address this gap by analyzing intra-city variations in BUD within Delhi and their relationship to urban well-being. Specifically, we examine how urban density correlates with indicators such as green space access, groundwater availability, and employment opportunities – elements we refer to collectively as urban well-being capitals. By leveraging ward-level data and integrating census information with remote sensing and spatial clustering techniques, this study elucidates how urban density both reflects and reproduces socio-economic inequalities.

Importantly, this study introduces the concept of Delhi’s ‘inverted compact’ form—a novel observation where population density is significantly higher in peripheral zones than in the urban cores, defying conventional urban models. This spatial reversal raises critical questions about planning, resource distribution, and infrastructure provision.

This study addresses the following research questions:

1. How does built-up density (BUD) relate to access to urban well-being indicators such as green space, groundwater availability and employment?
2. What spatial patterns of inequality emerge when clustering density with ecological and infrastructure indicators?
3. How can these patterns inform sustainable urban planning and zoning policies in Delhi?

To answer these questions, this study introduces a novel micro-scale analysis of BUD variations across neighborhoods by integrating multi-source datasets - including census and remote sensing data. It applies spatial-analytical approach to evaluate urban well-being in the study area. By addressing key research gaps in the existing literature, this study provides actionable insights for policymakers and urban planners to promote more inclusive and sustainable urban environments.

**Fig. 1:**
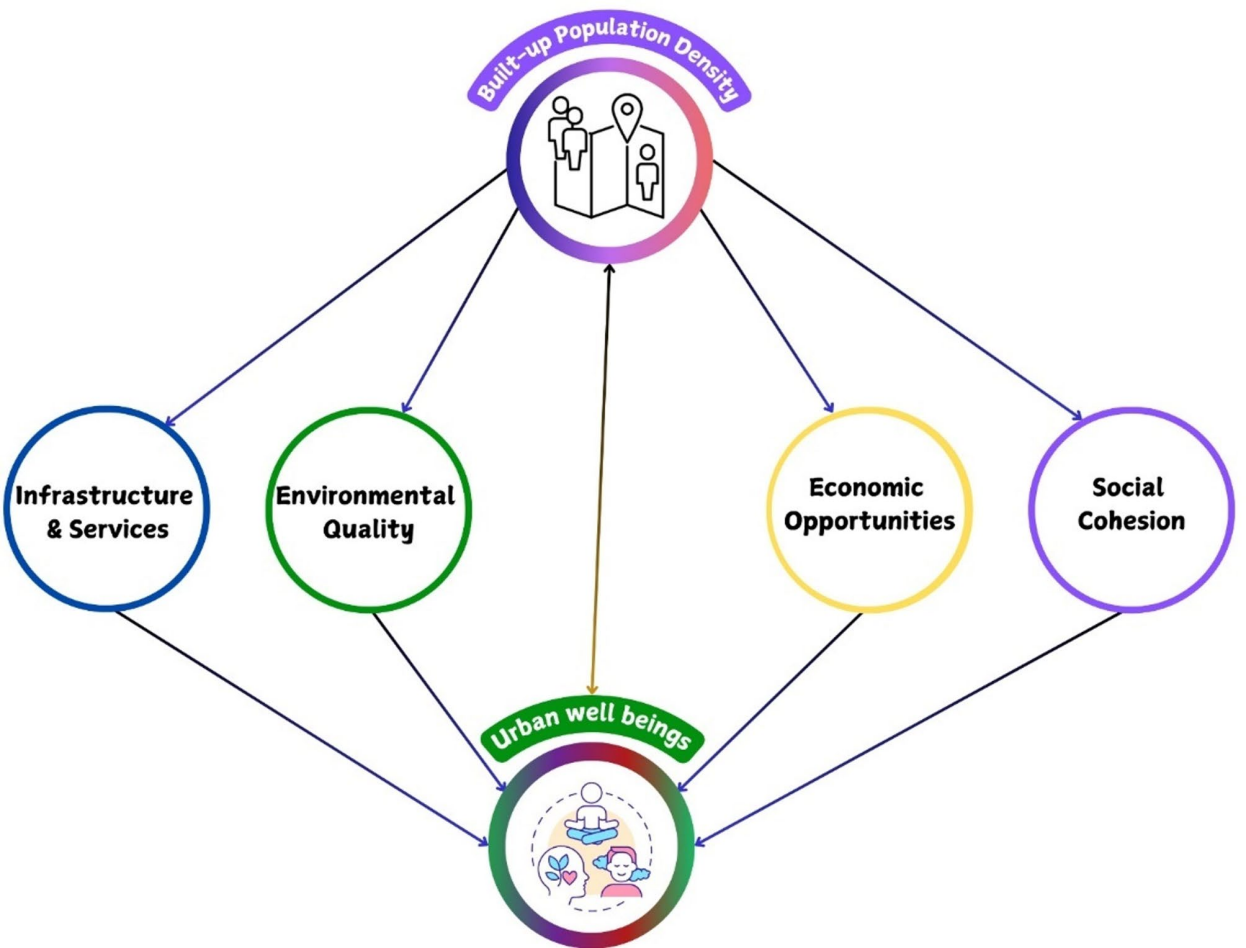
Conceptual framework of BUD and Urban well-being capitals.

The conceptual framework in Fig. 1 depicts interconnections between BUD, urban well-being capitals (including infrastructure access, environmental quality, and economic opportunity), and the dynamic nature of density across Delhi. This framework conceptualizes BUD as both a driver and an outcome of urban processes, shaped by governance structures, land use policies, and socio-economic conditions. It highlights a bidirectional relationship: while population density influences access to urban well-being resources, disparities in these indicators, in turn, shape density patterns through migration, development intensity, and land use change. By mapping these interactions, the framework provides a lens through which to understand how urban density impacts sustainability outcomes, particularly in relation to SDG 11, which promotes inclusive, safe, resilient and sustainable cities.

## Materials and methods

### Study area

**Fig. 2:**
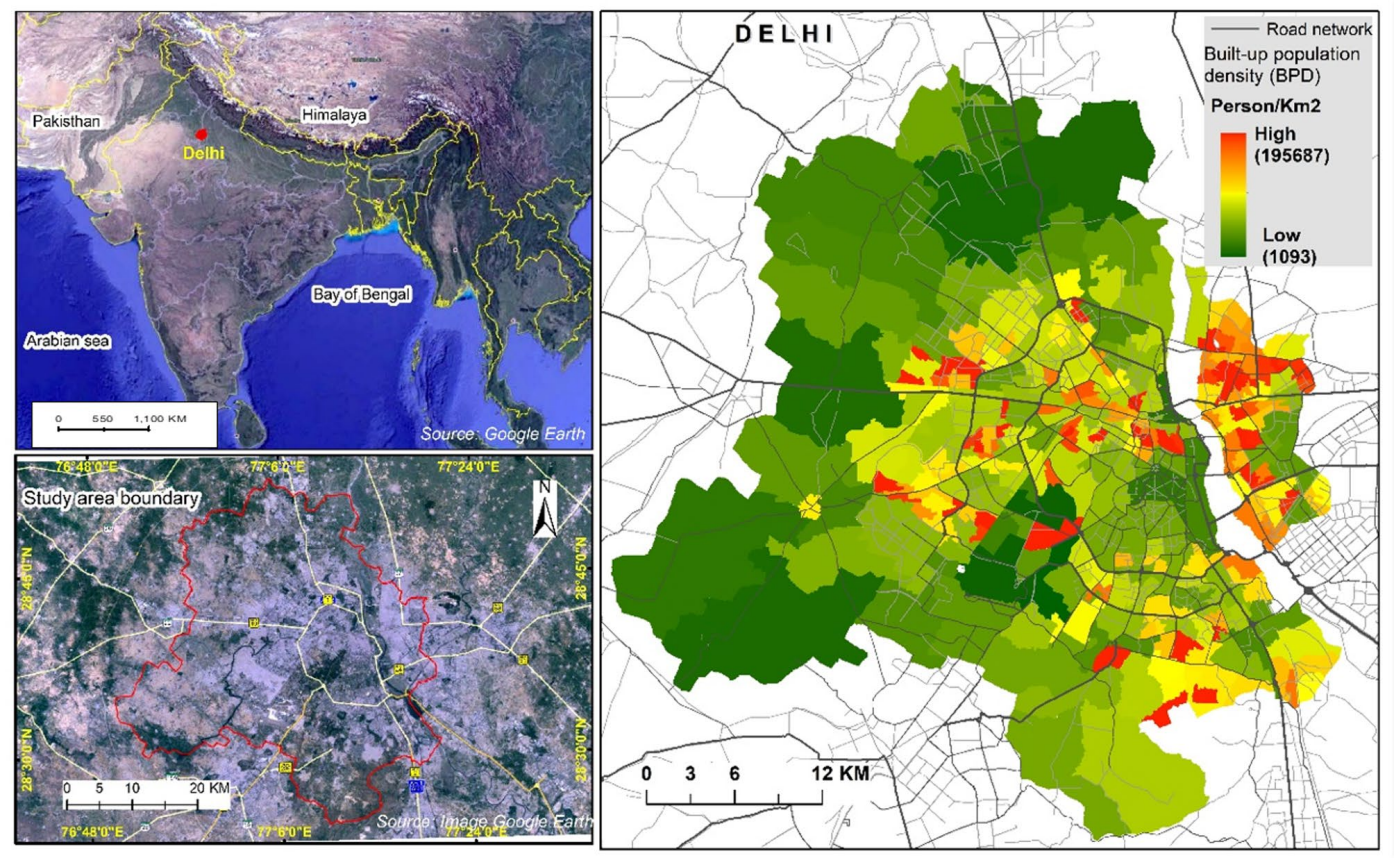
Map of the study area generated using ArcGIS Pro3.4 (licensed version https://pro.arcgis.com/en/pro-app/latest/get-started/download-arcgis-pro.htm). The map includes: (i) India with Delhi marked in red, (ii) Google Earth image of Delhi’s built-up area, (Map imagery obtained from ©Google Earth) and (iii) ward boundaries shaded by population density (green = low, red = high). Data source: https://diva-gis.org and https://censusindia.gov.in.

The city of Delhi, with an estimated population of 26 million and a built-up area of 1,212 km², ranks among the most populous and densely populated cities in South-Central Asia. As of 2015, its population density reached approximately 22,000 individuals per km²^12,13^. Delhi’s urban structure diverges from the conventional compact city model, instead of exhibiting an ‘inverted compact’ form, characterized by lower gross residential densities in the core and higher densities in peripheral region. The urban fabric of Delhi is a complex mosaic comprising planned low-rise colonial developments (e.g., Lutyens’ Delhi), modern high-rise residential areas (e.g., Dwarka, Rohini, and Narela), and a significant proportion of low-rise informal settlements and unregulated developments on previously undeveloped land (Fig. 2).

This study investigates intra-city variations in built-up density across diverse neighborhood types in Delhi, encompassing formally planned developments, informal high-rise clusters, and slums settlements. Kumar’s (2000) critique of Delhi as an inverted compact city, that Delhi, despite its high density, does not reap the typical benefits of compact cities – including efficient land and resource use, enhanced social interactions, shorter commutes and improves basic service delivery^14^. Delhi’s unique form presents a valuable opportunity to examine the relationship between urban density and human well-being. The aim is to contribute nuanced insights into how spatial patterns of density affect quality of life and inform sustainable and inclusive urban planning.

Urbanization has emerged as a significant global challenge, with land-use changes threatening environmental sustainability and ecosystem services (ESs)^15,16^. Evaluating the impact of urban expansion on ESs is vital for planners and policymakers. Bhuiyan et al. (2024) examined the spatiotemporal growth of Dhaka and found a substantial 68.3% decline in ESs from 2000 to 2021. The decline raises concerns about increased vulnerability to flooding, pollution and environmental degradation^16^. Similarly, Xu et al. (2019a) used the FUTURES model to simulate urban expansion scenarios in Beijing for 2035. They observed significant losses in ESs due to cropland conversion, although policy interventions helped mitigate these impacts. Their study highlights the necessity of optimized urban planning policies for achieving a balance between development and environmental preservation^17^.

Recognizing the uniqueness of Delhi’s urban dynamics, this study not only addresses local urban challenges but also situates them within a broader international context. Delhi was selected due to its rare ‘inverted compact’ urban form, pronounced intra-city disparities in infrastructure and environmental assets, and relevance to broader debates on urban form and sustainability. These features provide a compelling case for analyzing how urban density relates to human well-being at a micro scale.

### Methods

Two main data types were used: (i) ward-level socioeconomic data and (ii) remote sensing imagery (Landsat 8 and Google Earth). Spatial analysis was conducted in ArcGIS Pro 3.4, while R software was used for non-spatial data processing. ArcGIS was used to map BUD, identify spatial clusters and visualize well-being indicators. R tools were employed to compute correlations between BUD and well-being capitals.

Socioeconomic data from the 2011 Census of India provided ward-level information on household amenities and assets, such as building materials, water supply, drainage, and vehicle ownership. Remote sensing data (Landsat 8 OLI/TIRS, 2015) was used to extract environmental variables, including built-up area, surface imperviousness, land surface temperature (LST), and per capita green spaces. Per capita green space and groundwater levels were prioritized as well-being indicators due to their environmental and public health significance. Green space enhances urban livability by mitigating urban heat, improving air quality, and providing recreational opportunities. Groundwater, a critical resource in Delhi, faces a significant sustainability challenge due to over-extraction.

#### Environmental and socioeconomic variables

After initial variable selection, we computed correlations between BUD (dependent variable) and independent variables, including social capital, housing services, household assets, and environmental indicators (Table 1).

#### Geospatial data analysis

Landsat 8 OLI TIRS Level-1 data for March, 2015 was used to estimate built-up area, surface imperviousness, green space, and LST. A Support Vector Machine classifier was used for land cover classification, known for its high accuracy in urban settings^18^. Using the established methodologies, the impervious surface index and the Modified Normalized Difference Impervious Surface Index (MNDISI) were calculated to quantify the extent of impervious surfaces and extract impervious area, respectively^19^. Groundwater data from 115 borewells, sourced from the Central Ground Water Board (CGWB), were spatially interpolated using the Kriging method to generate a continuous groundwater surface. As a geostatistical interpolation technique, Kriging models spatial autocorrelation among measured points to produce statistically optimized surface predictions. This approach thus enables a more accurate assessment of groundwater variability across the study area. Correlation analysis was conducted to quantify associations between BUD and selected well-being indicators. This statistical validation further enhanced the credibility of spatial patterns identified through geospatial analysis.

### Cluster analysis

To decode spatial variations in BUD, we employed a threshold-based partitional clustering technique. This data-driven method segmented the city into analytically distinct clusters with similar density profiles, enhancing interpretability and minimizing intra-cluster variance. Unlike arbitrary administrative boundaries, this approach reflects actual population pressures and improves the robustness of our findings. This categorization is essential in a heterogeneous city like Delhi, where traditional zoning may not reflect real population pressures. By ensuring that comparisons are made between statistically distinct density regimes, the clustering approach adds methodological rigor and strengthens the reliability of policy-relevant insights derived from the study.

Six clusters were identified in ArcGIS through expert judgement, which allowed a targeted analysis of well-being indicators within each density regime. Spatial patterns were assessed in ArcGIS and R Studio, and correlation strengths were computed across clusters.

Field observations were conducted to validate the quantitative findings. For instance, Cluster 6 (Ajmeri Gate), with the highest BUD (136,385 persons/km²), was visually confirmed to be overcrowded with minimal open space Cluster 1 (South-West Delhi), with the lowest BUD (2,885 persons/km²), exhibited sparse development and abundant green space. These on-site confirmations are grounded in statistical results in lived urban conditions.

**Fig. 3:**
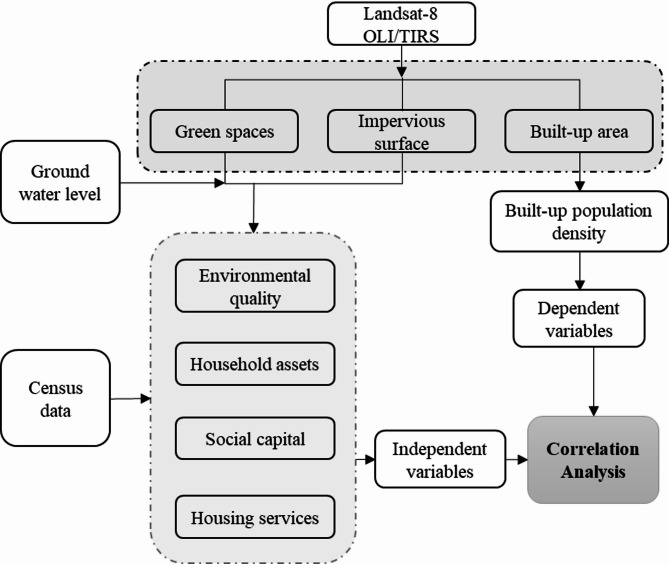
Analytical flowchart: built-up density and Urban well-being nexus.

The field-based observation technique complemented quantitative methods, capturing user behavior and physical interactions within neighborhoods. This mixed-methods approach allowed us to link spatial metrics with lived urban realities, ensuring our interpretations were both empirically sound and contextually grounded. The observation technique is a powerful tool for investigating the built environment and revisiting small details involved in users’ behaviors and interactions in the neighborhood. The overall steps of the materials and methods section are represented in Fig. 3, which details the analytical flowchart of BUD and well-being nexus, outlining each step from data collection through analysis and validation of our findings.

**Table 1:** Influential factors of urban built-up population density (BUD).

Dimension	Variables	Remarks
Human Capital	Literacy rate;	Education and income opportunities promote sustainable development at the local, regional, and national level social sustainability rely on employment opportunities for all and reducing income inequality of a society^20^.
	Main work;	
	Work participation	
Housing services	Tap water supply;	Drainage and sewage networks are crucial for sustainability in terms of health issues, urban flood management, and waste management. While the availability of an electricity connection alone is an incomplete measure of energy access, affordability and clean energy accessibility are crucial parts of the SDGs^21^.
	Sewerage system;	
	Indoor bath facility;	
	Drainage network	
Household assets	Bicycle;	Although private cars do not support sustainable transportation systems^22^, however, they reflect the financial status in developing countries. Sustainable urban development and human health and well-being would require walking and cycling as the best mode of transportation^23^.
	Scooter/motorbike;	
	Private car;	
Environmental capital	Per capita green;	The significance of environmental topologies such as green spaces, fresh water, surface imperviousness advocate sustainable urban development^24^.
	Groundwater level;	
	Impervious surfaces	

## Results

### Spatial variation of density and capitals

#### Built-up population density (BUD)

**Fig. 4:**
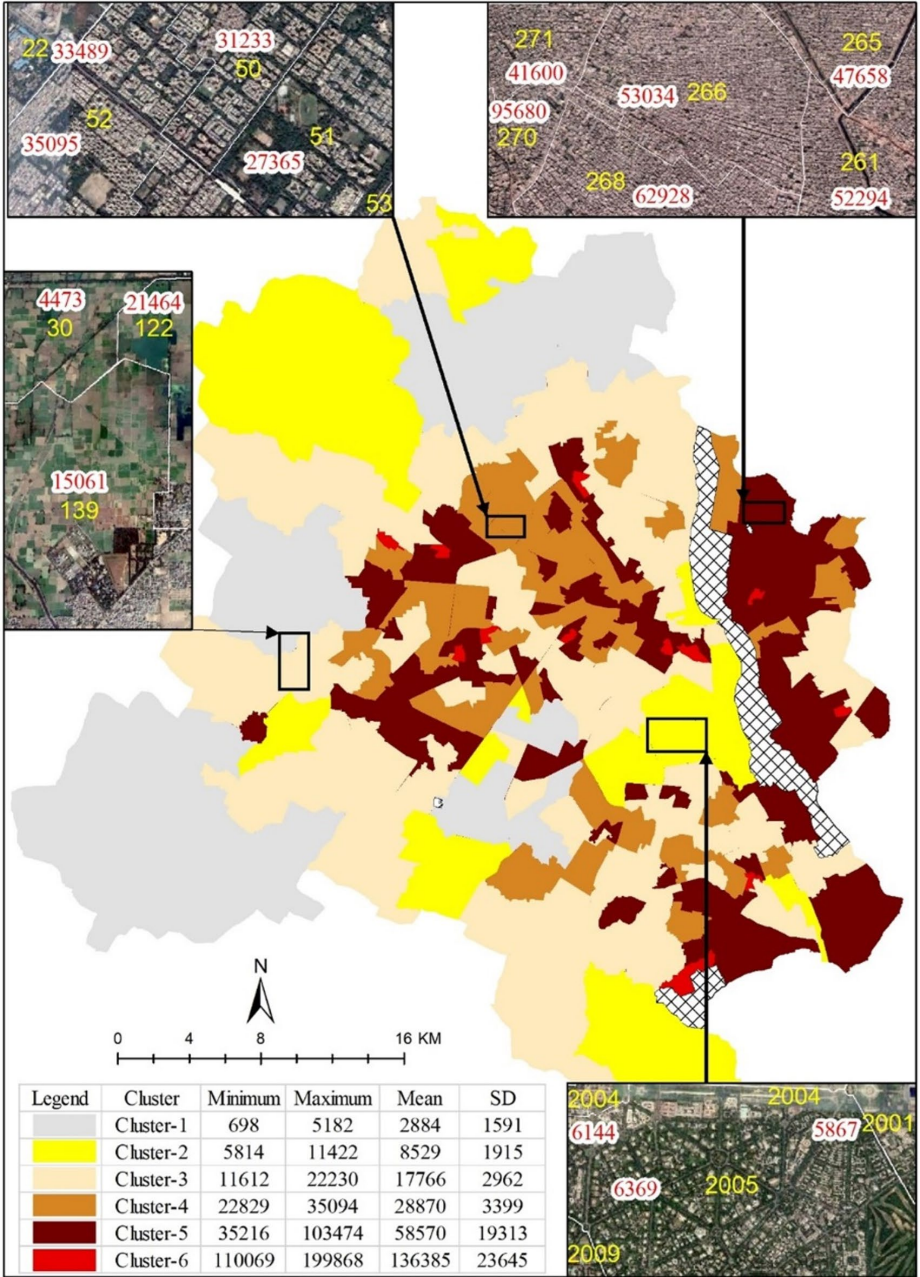
Spatial distribution of BUD clusters across Delhi generated using ArcGIS pro3.4 (licensed version) software and census data and google image *(*Map imagery obtained from ©Google Earth).

The density estimates were calculated using built-up areas derived from remote sensing data and census population data for each ward in Delhi. Figure 4 shows the spatial distribution of BUD clusters in the study area. The results revealed that the farmlands of South-west Delhi (Cluster-1) show a mean built-up population density of 2,884 persons/km^2^ (SD:1,591). This lower density is indicative of the predominantly agricultural land use and fewer urban structures in the area, therefore leading to lower concentration of population. On the opposite end of the spectrum, the highest built-up population density, 136,385 persons/km^2^, is in the neighborhood cluster of Old Delhi in the Ajmeri Gate area (Cluster-6). The considerably higher density in Old Delhi is a testament to its historical growth, urban sprawl, and the subsequent congestion. This finding underscores the intricate relationship between a city’s history, age, and its urban neighborhoods’ population density. Older neighborhoods, steeped in history, often exhibit higher population densities due to historical growth patterns. This phenomenon, in turn, necessitates tailored development strategies that recognize and address the unique needs and challenges posed by these denser and older areas. Intriguingly, the majority of the city’s population (0.63) resides in Cluster-5, an unplanned colony in Karawal Nagar located in East Delhi. This fact emphasizes the intricate challenges related to providing services and developing infrastructure in densely populated, yet unplanned, urban regions. Our findings reveal that Delhi’s current BUD has already surpassed the projected limits suggested in global studies in a significant portion (62%) of its wards. Furthermore, this study found that while Delhi’s gross population density stands at approximately 35,551 persons/km^2^, its BUD averaged approximately 43,671 persons/km^2^. The disparity between these two metrics signifies a high concentration of population in built-up areas, potentially leading to overcrowding, over-utilization of infrastructure, and a consequential strain on urban services.

**Fig. 5:**
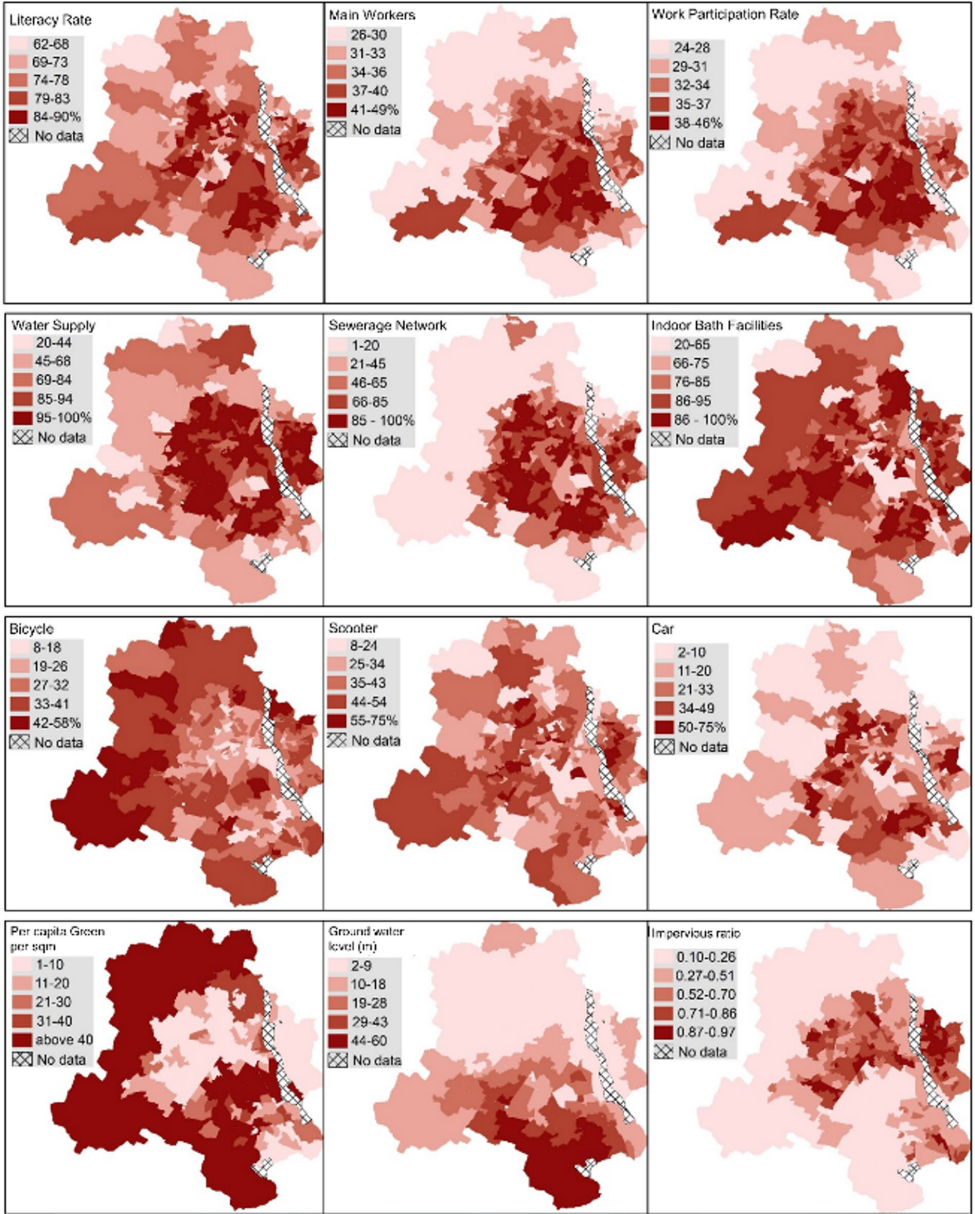
Geographic dispersion of well-being capitals across Delhi’s Urban landscape.

Per capita green spaces show considerable variation, with the average figure of 34 m^2^ dipping to around 10.6 m^2^ in wards without large reserve forests or plantations. This underlines the inequitable distribution of green spaces, with the majority of wards having access to limited green spaces. This unequal distribution could lead to environmental issues and might exacerbate the urban heat island effect in densely populated wards. An alarming finding is the high average impervious surface ratio (61%), which suggests a significant proportion of the city is covered with materials that prevent natural water infiltration, contributing to a higher risk of flooding and lower groundwater recharge. The groundwater level at an average of 16.38 m further suggests potential water scarcity issues in the future. Table 2 offers a comprehensive statistical summary of different well-being capitals, while Fig. 4 provides a spatial representation of selected variables across the city at the ward level. These capitals exhibit substantial intra-city variability, implying that the distribution of well-being across the city is not homogeneous but varies significantly from one ward to another. This variance underscores the multifaceted nature of urban well-being, illuminating the complexity of urban life within the city’s diverse neighborhoods (Fig. 5).

**Table 2:** Summary of household well-being capital.

Well-being capital	Mean	SD	Minimum	Maximum
Social capitals				
Literacy rate (%)	76.7	6.2	6.8	89.7
Main work (%)	34.1	4.0	26.1	48.6
Work participation (%)	32.4	3.9	23.7	46.1
Housing services				
Access to water supply (%)	84.5	19.3	1.00	100
Access to sewerage (%)	63.8	31.7	1.02	99.9
Indoor bath facilities (%)	86.8	13.9	24.3	99.9
Household assets				
Bicycle (%)	29.5	9.05	7.07	58.3
Scooter (%)	38.9	11.8	8.04	75.4
Car (%)	21.8	17.4	1.06	75.1
Environmental quality				
Per capita green spaces (m^2^)	23.52	59.73	0.001	563.80
Ground water level (m)	16.384	13.902	2.322	59.871
Impervious surface ratio	0.605	0.285	0.004	0.998

#### Correlation of density and capitals

**Fig. 6:**
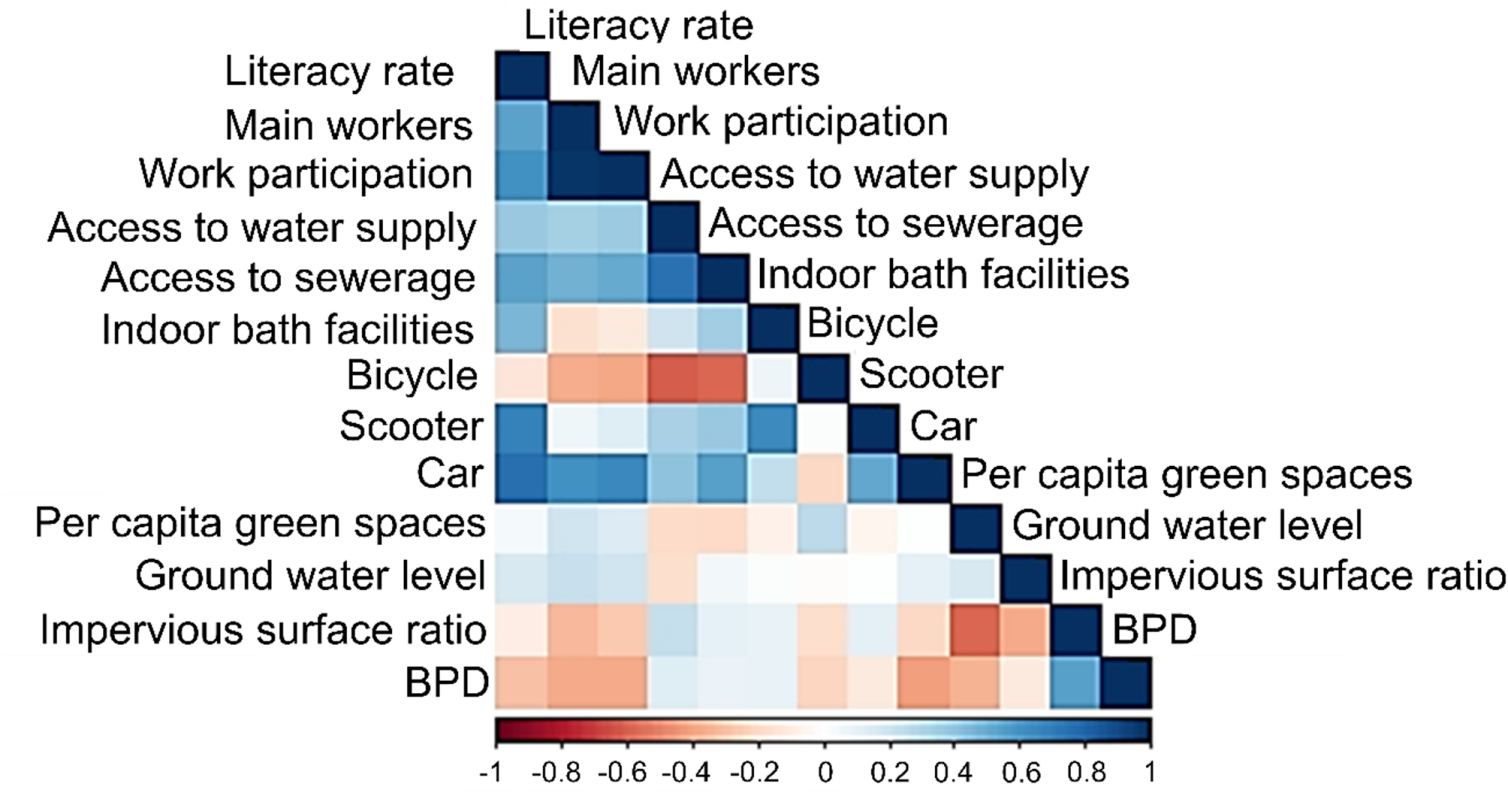
Interrelations among various forms of well-being capital: a ward-level correlation matrix. data source: census of India (2011). Note: BPD stands for built-up population density.

Figure 6 shows the correlation matrix to shed light on the relationships between built-up population density and various well-being capitals. Notably, wards with higher density might not necessarily correlate with higher access to services or assets, indicating potential disparities in service provision and living conditions in densely populated areas. These relationships require further investigation to inform the development of targeted urban policies.

### Comparative analysis of density clusters

This study identified six distinct clusters within Delhi using a dual-criteria approach based on both geographical proximity and socio-economic similarity. This methodology ensures that each cluster groups together wards that are not only spatially adjacent but also share comparable socio-economic conditions. Such clustering allows for a more meaningful comparison of urban challenges and well-being indicators across different parts of the city.

**Table 3:** Spatial and socio-economic definitions of the six Delhi clusters.

Cluster	Geographical zone	Socio-economic profile	Key characteristics
1	South-West Delhi	Low	Predominantly rural, agricultural land; low population density
2	Central Delhi	High	Commercial hubs, historical sites; high density and congestion
3	East Delhi	Medium	Mixed-use neighborhoods; moderate density; residential diversity
4	North Delhi	Medium–high	Residential zones with socio-economic diversity
5	West Delhi	Medium	Industrial-residential mix; varied density and infrastructure
6	South Delhi	High	Planned residential areas; relatively low density

A qualitative comparison of the urban development trends in the Delhi metropolitan area can be drawn from Tables 3 and 4. Spanning from the rural expanse of a South-West Delhi village (Cluster-1) to the dense, age-old urban neighborhoods in the Ajmeri Gate area (Cluster-6), these two clusters exhibit an urban-rural dichotomy. This dichotomy is demonstrated by the variation in ward-level mean density, which escalates from 2,885 inhabitants per km² in Cluster-1 to 65,833 inhabitants per km² in Cluster-6. Cluster-2, which encompasses the meticulously planned Lutyens’ Bungalow zone and the residential sector of Narela, showcases systematic development with generous green spaces and well-defined road networks. Conversely, Clusters-5 (Karawal Nagar) and − 6 (Ajmeri Gate) display haphazard, congested development patterns, underscoring the immense necessity for strategic planning in promoting urban harmony and environmental wellness. Labor force participation, a crucial measure of socio-economic well-being, varies markedly across the clusters. Cluster-2, with its central location and planned infrastructure, registers the maximum work participation rate of 35% (Table 4), thereby underscoring the positive correlation between well-planned infrastructure, locational advantage, and economic productivity. Conversely, Cluster-6, despite its dense population, records a lower work participation rate of 30%, pointing to the need for urban planning in boosting economic engagement.

Moreover, transportation facilities across the clusters present a wide range, reflecting the socio-economic variations. Cluster-1, predominantly rural, heavily relies on bicycle facilities, signifying the need for economic and sustainable transport modes in less affluent or sparsely populated regions. Conversely, Cluster-4, an affluent, well-planned neighborhood, boasts the most car parking facilities (34%), hinting at an association between socio-economic prosperity, infrastructure, and transportation choices. Environmental health, as denoted by green spaces per person and groundwater level, reveals sharp contrasts across clusters. Cluster-1, characterized by its sparse population and rural setup, offers the largest green space per person. Conversely, the densely populated, heavily urbanized Cluster-6 provides the least per person green space and has the lowest groundwater level, emphasizing the high environmental cost of unplanned urban densification. The distribution of well-being capital across these clusters highlights the advantages of planned development. Cluster-4, for instance, has the highest access to water supply and car facilities, suggesting superior living standards. In contrast, Cluster-6, with its high-density and lack of planning, suffers from significantly poorer access to resources and amenities, showcasing the adverse living conditions resulting from unregulated urban densification.

There is a striking variation in BUD across neighborhoods, particularly highlighting the sharp disparity between the centrally located Lutyens’ Bungalow zone (Cluster-2) and peripheral regions such as the village in South-west Delhi (Cluster-1). This compact urban form, with central areas having lower density and peripheral areas showing higher density, further affirms the complexity of urban planning. Notably, this analysis considered the differences in road networks and access to infrastructure across the clusters. The contrast between the well-organized road network in Cluster-2 (Lutyens’ Bungalow zone) and the disarray in Clusters 5 and 6 (Karawal Nagar and Ajmeri Gate area) highlights the need for comprehensive planning strategies in peripheral regions. Also, the variations in transportation facilities across the clusters are evident from Table 3. The analysis reveals higher car ownership in core areas like Cluster-4 (Pitampura) and higher bicycle ownership in the peripheries like Cluster-1 (South-west Delhi village), reinforcing the importance of infrastructure in shaping transportation choices.

**Table 4:** Summary of neighborhood cluster in Delhi based on density, urban form and well-being indicators.

Attribute	Cluster-1	Cluster-2	Cluster-3	Cluster-4	Cluster-5	Cluster-6
Location & Built Form	A village in South-west Delhi	Sample 2.1- Lutyens’ Bungalow zone located in the core-city of New Delhi, and 2.2 in the residential sector of Narela in the North	A planned group housing colony of Dwarka in West Delhi	A plotted colony of Pitampura in North Delhi	An unplanned colony of Karawal Nagar in East Delhi	A neighborhood cluster of old Delhi in the Ajmeri Gate area
Density (persons/km^2^)	2,885	8,529	17,766	28,870	58,570	136,385
Urban Characteristics	Scattered rural settlements; no structured pattern of road network	Type 1: Centrally located, well-planned areas with organized development, green spaces, and structured roads. Type 2: Emerging zones with mixed fabrics (rural and slums), limited green space, and mostly unplanned roads.	Mixed development; well-planned accessibility for planned areas with green coverages; heavily congested unplanned areas with high impervious surfaces.	Well-planned and block-based compact development; grid pattern of road network; enough green and public spaces	Unplanned and congested development pattern; no pattern of accessibility network; hardly any vegetation and open spaces; high impervious surfaces	Unplanned and congested informal settlement; no pattern of accessibility network; hardly any vegetation and open spaces; high impervious surfaces
Well-being indicators	Max green/person (225m^2^); min piped water (41%); max bicycle use (40%)	Max work participation rate (35%); Max main work (37%)	Max groundwater level (20 m)	Max piped water access (74%); Max car ownership (34%)	Max scooter use (41%); low main work (32%); average green space (4.55m^2^).	Min bicycle (22%); scooter (27%); car (6%); min green/person (0.35m^2^); lowest groundwater (12 m); lowest work participation rate (30%).

#### Research output-based specific policy interventions

Urban development strategies should endeavor to balance population density and quality of life. Table 5 shows cluster specific challenges and recommendations. Evident from Clusters 5 (Karawal Nagar) and 6 (Ajmeri Gate) is the fact that unregulated density can result in overcrowding and a significant strain on resources. However, well-structured, planned developments can host higher densities without compromising livability. Furthermore, equitable access to essential services and facilities serves as a vital indicator of the socio-economic health of neighborhoods. To this end, planners should work towards the equitable distribution of such amenities, particularly focusing on disadvantaged clusters such as Cluster-5 and Cluster-6 where resource accessibility is significantly limited.

**Table 5:** Cluster-Specific challenges and actionable Recommendations.

Cluster	Challenges identified	Recommendations
Cluster 1 - South-West Delhi Village	Low population density with inadequate infrastructure	Promote mixed-use development and improve public transport integration
Cluster 2 - Central Delhi (Lutyens’ Bungalow zone and Narela)	High density leading to congestion	Implement zoning restrictions, public transit upgrades, and congestion pricing
Cluster 3 - East Delhi (Mixed-use areas)	Moderate density with diverse residential and commercial use	Develop community spaces, enhance local transit
Cluster 4 - North Delhi (Residential with socio-economic diversity)	Socio-economic disparities affecting infrastructure	Launch targeted programs, upgrade infrastructure
Cluster 5 - West Delhi (Industrial and residential)	Industrial pollution and haphazard residential planning	Introduce green buffers and implement zoning reforms to manage land use
Cluster 6 - South Delhi (Highly planned residential areas)	Pressure on infrastructure due to planned high-density	Regulate building heights, increase green spaces

In high-density areas such as Cluster-6 (Ajmeri Gate), where congestion stems from overcrowded residential settlements and limited public infrastructure, we recommend the implementation of vertical zoning regulations that restrict additional residential floor area ratio (FAR) while encouraging ground-floor commercial use and upper-floor institutional development. This can help redistribute activity and reduce pressure on residential infrastructure. Urban design interventions such as pedestrianized streets, decentralized micro-markets, and multi-level parking structures can ease congestion and reclaim space for essential services. Furthermore, expanding Bus Rapid Transit (BRT) lanes along key corridors and introducing congestion pricing zones during peak hours would deter excessive vehicle use and incentivize public transport uptake.

In contrast, for low-density peripheral zones like Cluster-1 (South-West Delhi), mixed-use development is likely to be more effective than transport interventions alone. Rezoning policies should allow for integrated residential-commercial zoning to cluster housing, markets, and small businesses, which can generate local employment and reduce the need for long commutes. Once local density reaches a functional threshold, incremental metro extensions and improved last-mile connectivity can be introduced in phased to support future growth. This dual strategy aligns sustainable densification goals and enhances livability without replicating the congestion patterns of central zones.

The integration of green spaces into urban planning significantly enhances the quality of life in urban areas. Innovative strategies like rooftop gardens, vertical greening, and urban parks can be incorporated even in high-density areas, such as Cluster-6, to mitigate the environmental costs of intense urban densification. Importantly, in a city as diverse as Delhi, a one-size-fits-all strategy for urban development will not suffice. The city’s varied geographical and developmental contexts necessitate a more tailored, area-specific approach, considering the unique challenges and characteristics of each neighborhood cluster. As the city continues to urbanize, it becomes increasingly important to adopt a sustainable and inclusive approach to urban planning and development. This research provides critical insights that can guide policymakers and urban planners in developing strategies that not only improve living conditions but also work towards reducing socio-economic disparities across Delhi’s varied urban landscape. This balanced approach would pave the way for a more equitable and sustainable urban environment.

## Discussions

Urban BUD, a central topic in both academic and policy discourse, remains a pivotal concern as cities worldwide strive towards to achieve the SDGs^25^. Both of these international guidelines stress the importance of fostering urban well-being, a balance between high-density living and environmental sustainability, social livability, and economic efficiency^26^. Delhi’s urban morphology is complex, characterized by substantial variations in BUD. The city’s layout, a blend of meticulously planned regions, mixed-use commercial areas, and unplanned colonies, encapsulate the intricate dynamics of modern urban living. From planned residential spaces to unplanned settlements, a majority of Delhi’s residents inhabit an array of densities, exemplifying the city’s diverse urban fabric. In our investigation, we find that beyond a certain optimal neighborhood density, the specific economic activities within a location significantly determine its overall well-being^27^. Our analysis affirms the correlation between BUD and well-being capital, particularly in relation to amenities like water supply, green spaces, and vehicle ownership. Despite its significance, BUD emerges as one of many determinants of well-being capital^28^. This study enhances our understanding of how the urban built environment and density shape human well-being, and how socio-economic factors influence BUD, in particular^29^. This insight underscores the inherent complexity of urban living conditions, emphasizing the need for comprehensive urban planning and policymaking that integrates these multifaceted realities^30^. Such understanding aids in recognizing the critical roles that urban morphology and socio-economic factors play in shaping cities, providing an informed pathway towards sustainable urban development^31^.

The results reveal significant spatial variability in how BUD relates to well-being indicators, underscoring that a uniform policy response may not be effective. For instance, areas with high BUD but low green cover require immediate greening interventions and restrictions on further densification, whereas zones with low BUD and groundwater stress may benefit more from improved infrastructure and water conservation policies^32^. These spatial insights support zone-specific urban planning strategies—including tailored zoning regulations, infrastructure investments, and ecosystem restoration—aligned with localized environmental and socio-economic conditions. In general, such insights are invaluable for understanding the critical roles that urban morphology and socio-economic factors play in shaping cities, and they can effectively guide our efforts toward sustainable urban development. Furthermore, our research reveals the complex dynamics of Built-Up Development in the study area, weaving a rich narrative of urban realities with important implications for the city’s urban policy. The findings of this study provide insights for municipal authorities and service providers with the knowledge to customize the infrastructure delivery to the specific needs of diverse urban areas.

At a macroscopic level, this research can support policymakers, directing them towards areas that suffer from density extremes—those overlooked spots with minimal density and those peak points burdened by excessive density^33^. By acknowledging and rectifying these discrepancies, significant improvements can be achieved in neighborhood planning and consequently, the quality of urban life. Beyond diagnosing density-related challenges, this research also provides potential solutions. It highlights the pressing necessity for controlled densification in peripheral area, strategic re-densification of underutilized urban spaces^34^, and mindful redevelopment of deteriorating city cores. These interventions resonate with a growing body of research advocating for sustainable densification and redevelopment as pathways towards resilient and livable cities^35^.

This study underscores the significant environmental concerns associated with the increasing impervious surfaces in urban areas, particularly in high density urban neighborhoods. These surfaces disrupt the natural hydrological cycle by impeding groundwater recharge and increasing surface runoff, which contributes to both groundwater depletion and risk of urban floods during extreme rainfall events. Moreover, the accumulation of heat on impervious materials intensifies the Urban Heat Island (UHI) effect, thereby exacerbating thermal discomfort, energy demand for cooling, and public health vulnerabilities. To mitigate these cascading effects, urban planners should prioritize the adoption of Nature-based Solutions (NbS), including permeable pavements, green roofs, vegetated swales, and Sustainable Urban Drainage Systems (SUDS). Enhancing urban green cover and preserving natural hydrological pathways are critical strategies for improving stormwater regulation and strengthening urban resilience to climate-induced stressors such as heatwaves and flash floods.

Delhi’s per capita green space, estimated at approximately 20 m²^36^, remains below global recommendations. In several wards green space availability falls significantly short of the World Health Organization (WHO)’s minimum recommendation of 9 m² per capita for healthy urban living^24^. Moreover, the United Nations (UN) has identified a per capita minimum threshold of 30 m² of accessible green space^37^. In contrast, Delhi’s fragmented and uneven distribution of green infrastructure not only reinforces spatial inequities in informal or high-density settlements but also intensifies the urban heat island (UHI) effect, increases ecological vulnerability, and limits access to essential ecosystem services^32,38^.

To align with international standards and address the ecological and social implications of this deficit, the expansion of green infrastructure is needed. Key interventions include increasing the coverage and accessibility of urban parks, promoting urban forestry and tree canopy development, adopting green roofs and walls, and safeguarding natural drainage systems and vegetated open spaces. These NbS are increasingly recognized for their role in enhancing urban liveability, reducing flood risks, mitigating heat stress, and promoting equity in ecosystem service distribution^39,40^.

The implications of our findings extend well beyond Delhi city. The spatial patterns and socio-environmental insights revealed from this study have broader relevance for rapidly urbanizing regions, particularly in developing countries where cities face challenges such as population density, infrastructure stress, and ecological degradation. In this regard, this study serves not only as a detailed examination of Delhi’s urban morphology but also provides a framework for urban planners in other rapidly developing cities. This collective knowledge can be used to formulate data-driven planning strategies towards more inclusive, sustainable, and resilient urban development^41^. The heterogeneous density profiles across Delhi highlight the necessity for differentiated, zone-specific policy interventions. For instance, peripheral villages with low density should prioritize policies focused on providing basic infrastructure and promoting controlled densification, to ensure livability and affordability. In contrast, moderate-density areas, such as planned group housing colonies, should promote mixed-use development to reduce dependence on private vehicles and encourage non-motorized transportation. High-density, unplanned areas, like Karawal Nagar and Ajmeri Gate, require targeted interventions aimed at reducing congestion, enhancing infrastructure, and providing access to green spaces and public services.

Overall, the complex urban environment of Delhi, characterized by a wide spectrum of densities, calls for equally diverse and context-sensitive policy responses. The specific challenges and opportunities inherent in each cluster highlight the importance of adaptive, place-based urban governance strategies towards sustainable urbanization^35^. While this study offers a comprehensive analysis of urban BUD and well-being in Delhi, it is important to consider its limitations. First, reliance on ward-level data presents a notable limitation. While such data provides broad insights about urban density, it may not necessarily capture the detailed intra-ward variations in both density and well-being capital^42^. This may lead to oversights in identifying pockets of variance that could be hidden within the wards. Secondly, the density dynamics and their impact on well-being in Delhi might not mirror those in other urban areas, both within and beyond India. Thirdly, this study employs correlational analysis to explore the relationships between built-up density (BUD) and well-being capital. This method is effective at identifying patterns and links, but it cannot establish cause-and-effect relationships between variables^38,43^. Fourthly, while the well-being indicators used in this study are multidimensional—capturing aspects such as education, health, and asset ownership—they remain incomplete. They may not cover all aspects of well-being in each neighborhood, offering scope for further research. In addition, there is a temporal lag in socio-economic data because 2011 census was utilized. Addressing this gap calls for greater integration of mixed-methods approaches in future research. Participatory GIS (PGIS) tools—including community mapping workshops, photovoice methods, mobile applications for geo-tagging environmental stressors, and resident-led surveys—can enrich spatial datasets between census intervals. These limitations could be addressed by future studies using a long-term approach to track changes over time, providing deeper insight into how the relationship between urban density and well-being evolves and applications Geographically Weighted Regression (GWR) based analysis.

## Conclusions

This study provides a comprehensive analysis of built-up population density (BUD) and its complex relationship with human well-being in the urban context of Delhi using multi-source data. It identifies a distinctive urban form—an inverted compact city—characterized by spatially uneven population densities and pronounced socio-economic disparities. These findings challenge traditional urban models and highlight the need for more spatially adaptive planning.

While optimal neighborhood density contributes to improved well-being, our results indicate that its positive influence diminishes beyond a certain threshold. In high-density settings, socio-economic factors and the nature of economic activity play a more critical role in shaping well-being outcomes. This highlights the importance of integrated urban planning approaches that consider physical density alongside economic, social, and environmental dimensions.

To manage urban sprawl and promote equitable development, peripheral areas require planned densification strategies, including development caps, mixed-use zoning, and balanced expansion of residential, commercial, and public infrastructures. Spatially targeted investments must prioritize equitable access to healthcare, education, green spaces, and public transit. In low-density zones, densification can be encouraged through measures such as reduced development fees, expedited building approvals, and tax rebates for affordable housing, and enhanced transit connectivity – including Bus Rapid Transit (BRT) and last-mile linkages. In high-density areas, targeted interventions such as green infrastructure (e.g., rooftop gardens, vertical greening), enhancement of ventilation corridors, and the expansion of public green spacescan help improve livability and mitigate urban heat island effects.

These context-specific interventions support balanced urban growth, socio-spatial equity, and environmental resilience. The study emphasizes the importance of tailored urban planning policies to the specific challenges and opportunities presented by low, moderate and high-density neighborhoods. Strategic interventions – such as redevelopment of declining urban cores, re-densification of underutilized spaces, and sustainable expansion in peripheral areas – will be essential for creating more livable and inclusive cities.

Future research should adopt longitudinal designs to track how BUD and well-being indicators evolve over time. Monitoring changes in groundwater levels, per capita green space, and infrastructure access alongside changes BUD metrics may help identify critical thresholds for urban sustainability. Integrating qualitative methods, such as semi-structured interviews, focus groups, and participatory GIS, can also provide deeper insights into residents’ lived experiences and adaptive strategies in response to urban change. It supports the advancement of the SDG 11 – by outlining a data-informed pathway towards more inclusive, safe, resilient, and sustainable urban development.

## Data Availability

The datasets generated and/or analyzed during the current study are available from the corresponding author upon reasonable request.

## References

[1] Avtar, R., Aggarwal, R., Kharrazi, A., Kumar, P. & Kurniawan, T. A. Utilizing Geospatial information to implement SDGs and monitor their progress. *Environ. Monit. Assess.* **192** (1), 35. 10.1007/s10661-019-7996-9 (Dec. 2019). 10.1007/s10661-019-7996-9 31828438

[2] Ahmad, S., Avtar, R., Sethi, M. & Surjan, A. ‘Delhi’s land cover change in post transit era’, *Cities*, vol. 50, pp. 111–118, Feb. (2016). 10.1016/j.cities.2015.09.003

[3] Abubakar, I. R. & Aina, Y. A. The prospects and challenges of developing more inclusive, safe, resilient and sustainable cities in Nigeria. *Land. Use Policy*. **87**, 104105. 10.1016/j.landusepol.2019.104105 (Sep. 2019).

[4] Güneralp, B. et al. Aug., ‘Global scenarios of urban density and its impacts on building energy use through 2050’, *Proceedings of the National Academy of Sciences*, vol. 114, no. 34, pp. 8945–8950, (2017). 10.1073/pnas.1606035114 10.1073/pnas.1606035114 PMC5576775 28069957

[5] Roy, S., Bose, A., Basak, D. & Chowdhury, I. R. ‘Towards sustainable society: the sustainable livelihood security (SLS) approach for prioritizing development and understanding sustainability: an insight from West Bengal, India’, *Environ Dev Sustain*, vol. 26, no. 8, pp. 20095–20126, Aug. (2024). 10.1007/s10668-023-03456-x

[6] Majumder, S., Roy, S., Bose, A. & Chowdhury, I. R. Multiscale GIS based-model to assess urban social vulnerability and associated risk: evidence from 146 urban centers of Eastern India. *Sustainable Cities Soc.* **96**, 104692. 10.1016/j.scs.2023.104692 (Sep. 2023).

[7] Salem, M. et al. Jan., ‘Urban expansion simulation based on various driving factors using a logistic regression model: Delhi as a case study’, *Sustainability*, vol. 13, no. 19, Art. no. 19, (2021). 10.3390/su131910805

[8] Roy, S., Bose, A., Singha, N., Basak, D. & Chowdhury, I. R. ‘Urban waterlogging risk as an undervalued environmental challenge: An Integrated MCDA-GIS based modeling approach’, *Environmental Challenges*, vol. 4, p. 100194, Aug. (2021). 10.1016/j.envc.2021.100194

[9] Roy, S. et al. ‘Evaluating urban environment quality (UEQ) for Class-I Indian city: an integrated RS-GIS based exploratory Spatial analysis’. *Geocarto International*, **38**, 1, p. 2153932, 10.1080/10106049.2022.2153932

[10] Trossman-Haifler, Y. & Fisher-Gewirtzman, D. ‘How urban wellbeing is influenced by spatial urban parameters (density, morphology, vegetation & commerce), as examined in a VR framework’, *Architectural Science Review*, vol. 65, no. 5, pp. 370–384, Sep. (2022). 10.1080/00038628.2022.2091510

[11] Roy, S., Majumder, S., Bose, A. & Chowdhury, I. R. ‘GWPCA-based spatial analysis of urban vitality: a comparative assessment of three high-altitude Himalayan towns in India’, *Journal of Spatial Science*, vol. 69, no. 2, pp. 593–620, Apr. (2024). 10.1080/14498596.2023.2267011

[12] Singh, S. et al. ‘Identifying micro-level pollution hotspots using sentinel-5P for the spatial analysis of air quality degradation in the national capital region, India’, *Sustainability*, vol. 17, no. 5, Art. no. 5, Jan. (2025). 10.3390/su17052241

[13] Florczyk, A. et al. ‘Description of the GHS Urban Centre Database 2015’, JRC Publications Repository. Accessed: Feb. 18, 2025. [Online]. Available: https://publications.jrc.ec.europa.eu/repository/handle/JRC115586

[14] Kumar, A. *The Inverted Compact City of Delhi’, in Compact Cities* (Routledge, 2000).

[15] Misra, P. et al. Mapping brick kilns to support environmental impact studies around Delhi using Sentinel-2. *ISPRS Int. J. Geo-Information*. **9**, 10.3390/ijgi9090544 (Sep. 2020). 9, Art. 9.

[16] Bhuiyan, M. A. H. et al. The differential impacts of the Spatiotemporal vertical and horizontal expansion of megacity Dhaka on ecosystem services. *Curr. Res. Environ. Sustain.* **7**, 100252. 10.1016/j.crsust.2024.100252 (Jan. 2024).

[17] Xu, Q., Zheng, X. & Zheng, M. Do urban planning policies Meet sustainable urbanization goals? A scenario-based study in beijing, China. *Sci. Total Environ.* **670**, 498–507. 10.1016/j.scitotenv.2019.03.128 (Jun. 2019). 10.1016/j.scitotenv.2019.03.128 30904661

[18] Pal, M. & Mather, P. M. ‘Support vector machines for classification in remote sensing’, *International Journal of Remote Sensing*, vol. 26, no. 5, pp. 1007–1011, Mar. (2005). 10.1080/01431160512331314083

[19] Sun, Z., Wang, C., Guo, H. & Shang, R. ‘A modified normalized difference impervious surface index (MNDISI) for automatic urban mapping from landsat imagery’, *Remote Sensing*, vol. 9, no. 9, Art. no. 9, Sep. (2017). 10.3390/rs9090942

[20] Mondal, B. & ‘Pandemic, COVID-19, reduced usage of public transportation systems and urban environmental challenges: few evidences from India and West bengal’. In *Environmental Management and Sustainability in India: Case Studies from West Bengal* (eds Sahu, A. S. & Das Chatterjee, N.) 341–368 (Springer International Publishing, 2023). 10.1007/978-3-031-31399-8_16.

[21] Bibi, T. S., Reddythta, D. & Kebebew, A. S. ‘Assessment of the drainage systems performance in response to future scenarios and flood mitigation measures using stormwater management model’, *City and Environment Interactions*, vol. 19, p. 100111, Aug. (2023). 10.1016/j.cacint.2023.100111

[22] Steg, L. ‘Sustainable transportation: a psychological perspective’, *IATSS Research*, vol. 31, no. 2, pp. 58–66, Jan. (2007). 10.1016/S0386-1112(14)60223-5

[23] Tight, M. ‘Sustainable urban transport – the role of walking and cycling’, *Proceedings of the Institution of Civil Engineers - Engineering Sustainability*, vol. 169, no. 3, pp. 87–91, Jun. (2016). 10.1680/jensu.15.00065

[24] Edeigba, B. A., Ashinze, U. K., Umoh, A. A., Biu, P. W. & Daraojimba, A. I. Urban green spaces and their impact on environmental health: A global review. *World J. Adv. Res. Reviews*. **21** (2), 917–927. 10.30574/wjarr.2024.21.2.0518 (2024).

[25] Keith, M. et al. Feb., ‘A new urban narrative for sustainable development’, *Nat Sustain*, vol. 6, no. 2, pp. 115–117, (2023). 10.1038/s41893-022-00979-5

[26] Zhang, K. & Yan, D. ‘Exploring indoor and outdoor residential factors of high-density communities for promoting the housing development’, *Sustainability*, vol. 15, no. 5, Art. no. 5, Jan. (2023). 10.3390/su15054452

[27] Wu, J. ‘Landscape sustainability science: ecosystem services and human well-being in changing landscapes’, *Landscape Ecol*, vol. 28, no. 6, pp. 999–1023, Jul. (2013). 10.1007/s10980-013-9894-9

[28] Kassouri, Y. & Alola, A. A. Towards unlocking sustainable land consumption in sub-Saharan africa: analysing spatio-temporal variation of built-up land footprint and its determinants. *Land. Use Policy*. **120**, 106291. 10.1016/j.landusepol.2022.106291 (Sep. 2022).

[29] Mouratidis, K. ‘Compact city, urban sprawl, and subjective well-being’, *Cities*, vol. 92, pp. 261–272, Sep. (2019). 10.1016/j.cities.2019.04.013

[30] Bibri, S. E. Data-driven smart eco-cities and sustainable integrated districts: A best-evidence synthesis approach to an extensive literature review. *Eur. J. Futures Res.* **9** (1), 16. 10.1186/s40309-021-00181-4 (Nov. 2021).

[31] Sapena, M. & Ruiz, L. A. Identifying urban growth patterns through land-use/land-cover spatio-temporal metrics: simulation and analysis. *Int. J. Geogr. Inf. Sci.* **35** (2), 375–396. 10.1080/13658816.2020.1817463 (Feb. 2021).

[32] Kumar, M. et al. ‘Case study 4: urban green space analysis and potential site selection for green space expansion in NCT delhi’, in Geographic Information Systems in Urban Planning and Management, (eds Kumar, M., Singh, R. B., Singh, A., Pravesh, R., Majid, S. I. & Tiwari, A.) Singapore: Springer Nature, 191–203. doi: 10.1007/978-981-19-7855-5_12. (2023).

[33] Wang, Z. et al. Impact of rapid urbanization on the threshold effect in the relationship between impervious surfaces and water quality in shanghai, China. *Environ. Pollut*. **267**, 115569. 10.1016/j.envpol.2020.115569 (Dec. 2020). 10.1016/j.envpol.2020.115569 33254687

[34] Scheba, A., Turok, I. & Visagie, J. ‘Inequality and urban density: socio-economic drivers of uneven densification in cape town’, *Environment and Urbanization ASIA*, vol. 12, p. 097542532199802, Mar. (2021). 10.1177/0975425321998026

[35] Wilkes-Allemann, J. et al. Jun., ‘Envisioning the future—Creating sustainable, healthy and resilient BioCities’, *Urban Forestry & Urban Greening*, vol. 84, p. 127935, (2023). 10.1016/j.ufug.2023.127935

[36] Shahfahad, B. et al. ‘Assessment of public open spaces (POS) and landscape quality based on per capita POS index in Delhi, India’, *SN Appl. Sci.*, vol. 1, no. 4, p. 368, Mar. (2019). 10.1007/s42452-019-0372-0

[37] Şenik, B. & Uzun, O. ‘A process approach to the open green space system planning’, *Landscape Ecol Eng*, vol. 18, no. 2, pp. 203–219, Apr. (2022). 10.1007/s11355-021-00492-5

[38] Tzoulas, K. et al. Jun., ‘Promoting ecosystem and human health in urban areas using Green Infrastructure: A literature review’, *Landscape and Urban Planning*, vol. 81, no. 3, pp. 167–178, (2007). 10.1016/j.landurbplan.2007.02.001

[39] Jha, P., Joy, M. S., Yadav, P. K., Begam, S. & Bansal, T. Detecting the role of urban green parks in thermal comfort and public health for sustainable urban planning in Delhi. *Discov Public. Health*. **21** (1), 236. 10.1186/s12982-024-00368-7 (Dec. 2024).

[40] ‘India-forum-. for-Nature-based-solutions-annual-summit-26th-Nov-WRI-Version.pdf’. Accessed: Jul. 11, 2025. [Online]. Available: https://wri-india.org/sites/default/files/India-forum-for-Nature-based-Solutions-annual-summit-26th-Nov-WRI-Version.pdf

[41] Groulx, M., Kieta, K., Rempel, M., Horning, D. & Gaudreau, K. ‘Smart growth in Canada’s provincial north’, *Planning Practice & Research*, vol. 37, no. 2, pp. 231–247, Mar. (2022). 10.1080/02697459.2021.1979786

[42] Bansal, B. ‘Generic neighborhood features of an egalitarian city: The postwar Tokyo Model’, *Cities*, vol. 131, p. 103920, Dec. (2022). 10.1016/j.cities.2022.103920

[43] Gao, K., Yang, Y., Gil, J. & Qu, X. ‘Data-driven interpretation on interactive and nonlinear effects of the correlated built environment on shared mobility’, *Journal of Transport Geography*, vol. 110, no. C, Accessed: Feb. 18, 2025. [Online]. (2023). Available: https://ideas.repec.org//a/eee/jotrge/v110y2023ics0966692323000765.html

